# Using systematic observations to understand conditions that promote inter-racial experiences in neighbourhood parks

**DOI:** 10.17645/up.v1i4.756

**Published:** 2016-12-09

**Authors:** Amy Hillier, Bing Han, Theodore S. Eisenman, Kelly R. Evenson, Thomas L. McKenzie, Deborah A. Cohen

**Affiliations:** Department of City & Regional Planning, School of Design, University of Pennsylvania, USA; RAND Corporation, Santa Monica, USA; Department of Landscape Architecture and Regional Planning, University of Massachusetts, USA; Department of Epidemiology, Gillings School of Global Public Health, University of North Carolina—Chapel Hill, USA; Institute for Behavioral and Community Health, School of Exercise and Nutritional Sciences, San Diego State University, USA; RAND Corporation, Santa Monica, USA

**Keywords:** Intergroup contact theory, urban parks, interracial contact, SOPARC, parks and recreation

## Abstract

We analysed observations from 31 neighbourhood parks, with each park mapped into smaller target areas for study, across five US cities generated using the System for Observing Play and Recreation in the Community (SOPARC). In areas where at least two people were observed, less than one-third (31.6%) were populated with at least one white and one non-white person. Park areas that were supervised, had one or more people engaged in vigorous activity, had at least one male and one female present, and had one or more teens present were significantly more likely to involve interracial groups (p<0.01 for each association). Observations in parks located in interracial neighbourhoods were also more likely to involve interracial groups (p<0.05). Neighbourhood poverty rate had a significant and negative relationship with the presence of interracial groups, particularly in neighbourhoods that are predominantly non-white. Additional research is needed to confirm the impact of these interactions. Urban planning and public health practitioners should consider the health benefits of interracial contact in the design and programming of neighbourhood parks.

## 1. Introduction

Urban parks have held a prominent place in city planning, landscape architecture, and public health scholarship for well over a century (Cranz 1982; Wheater et al 2015). Recent literature has identified four potential pathways whereby green space in cities may promote public health: stress reduction, increased physical activity, improved air quality, and social cohesion (Hartig et al. 2014). The first three of these pathways have received more scholarly attention than the democratic and social implications of people across race/ethnicity and socioeconomic status coming together in public spaces (Eisenman 2016). Building from the concept of psychosocial health, this paper considers the role of parks in bringing people together across racial/ethnic groups and, potentially facilitating interracial contact as an important and underappreciated pathway to increasing social cohesion, reducing racial prejudice, and improving human health. We analyse observations from 31 neighbourhood parks in five US cities to determine the characteristics of parks, neighbourhoods in which the park is located, and activities offered at the park that correlate with people across racial groups simultaneously occupying the same part of the park. We conclude by calling on urban design and planning to focus on creating and managing public spaces that promote social interaction across race/ethnicity as well as income, gender, and age groups.

Before describing data collection and analysis methods, we review literature from four distinct areas of scholarship that together create the conceptual and methodological foundation for our research: (1) urban planning and landscape architecture’s history of promoting urban parks as democratic public spaces that foster cohesion between groups of different socio-economic and ethnic background; (2) research on social interaction, social cohesion, and intergroup contact in public spaces and green spaces within leisure studies and environmental and social psychology; (3) public health research on chronic exposure to prejudice and institutional racism as primary contributors to racial health disparities; and (4) recent public health research utilizing systematic social observations and environmental audits of outdoor public spaces including neighbourhood parks. By borrowing from these distinct areas of scholarship, we present a conceptual framework and suggestions for measurement and research design that highlight and test the underappreciated public health benefits of people coming together across race and ethnicity in neighbourhood parks.

## 2. Background and Significance

### 2.1. Urban Parks

As the world undergoes a third major period of urbanization (Angel 2011), local governments are adopting new types of parks and green space strategies. This includes creation of rail trails and greenways, retrofitting landfills, cemeteries, rooftops, and parking areas, covering highways and reservoirs, sharing schoolyards, closing roads, and creating urban farms and community gardens (Harnik 2010). There is also increasing attention on tree planting and site scale greening initiatives (Keeley 2011; Young 2011). Situated within an historical context, this bloom of activity can be seen as an effort to increase the liveability of cities in an urbanizing age (Eisenman 2016), in much the same way that reform-minded urban designers and leaders advanced city parks in the 19^th^ century (Schuyler 1986).

Historically, social workers, urban planners, landscape architects and public health practitioners looked to parks as antidotes to many of the problematic and unhealthy aspects of cities (Dannenberg, Frumkin & Jackson 2011). The 19^th^ century parks movement developed in response to the negative impact of urban industrialization on physical health, mental health, and social bonds (Cranz 1982; Schuyler 1986; Eisenman 2013). Frederick Law Olmsted, Sr. the 19^th^ century landscape architect famous for designing Manhattan’s Central Park, Brooklyn’s Prospect Park, and Boston’s Emerald Necklace, thought natural scenery was critical “to give the mind a suggestion of rest from the devouring eagerness and intellectual strife of town life” (Olmsted 1871). Olmsted believed that parks would promote democratic values and social life by bringing together diverse people, “each individual adding by his mere presence to the pleasure of all others” (Olmsted 1871).

The large, curvilinear “pleasure grounds” of the 19^th^ century that benefited primarily upper middle class residents gave way to the smaller, rectilinear “reform parks” of the early 20^th^ century, focused on social reform, children’s play, and assimilation of European immigrants (Cranz 1982; Cranz & Boland 2004). During the Progressive Era, parks were expected to “reduce class conflict, reinforce the family unit, to socialize immigrants to the American way of life, to stop the spread of disease, and to educate citizens” (Cranz & Boland 2004, 103). During the mid-20^th^ century, parks became recognized primarily as sites of recreation, and stadiums and asphalt basketball courts were added liberally. By the 1960s, some public officials looked to parks to help resolve racial tensions and stop riots, focusing on open space as places of participation, revitalization, and social control (Cranz 1982). But as with Olmstead Sr.’s hope that the mere presence of diverse people together in green spaces would add to the “pleasure of others,” these efforts to reduce racial tensions lacked strong theoretical foundations and empirical evidence.

### 2.2. Social interaction and intergroup contact

Urban parks have been associated with positive mental health benefits distinct from any physical health benefits such as increased physical activity (Sturm & Cohen 2014; Sugiyama et al. 2008). Public health studies have shown a correlation between access and use of parks or open space and lower resting heart rate, reduced stress, and better mental health across age groups (Bilseviciene et al 2014; Grazuleviciene et al 2014; Song et al 2014). Much of the literature linking use of public spaces to health focused on the restorative nature of green settings and contact with nature (Kaplan & Kaplan 1969; Ulrich 1989; Francis et al 2012 SSM). Less research has focused on social interaction as the important mechanism, with parks and open space facilitating the development of supportive relationships (Francis et al 2012 SSM; Francis et al 2012 JEP; Cattell et al 2008; Putnam2000; Berkman and Glass 2000). Public health research has considered racial/ethnic variation in park use (Derose et al 2015;), but these studies do not consider whether people across race/ethnicity are interacting in parks. Contemporary urban greening literature also addresses social cohesion – the reciprocity and trustworthiness that arise from social networks – as a possible link between urban green space and human health. Here, research suggests that community green spaces that do not impede ground level views can reduce antisocial outcomes such as crime and household aggression (Kuo and Sullivan 2001a, 2001b; Donovan and Prestemon 2012), and that this may be due to signalling social ties, increasing informal surveillance, or mitigating mental fatigue (Wilson & Kellling 2004; Jacobs 1961; Newman 1972; Kaplan 1995). Some studies also suggest that community green spaces can promote pro-social outcomes such as greater neighbourhood social ties, more face-to-face contact, larger groupings of people, and increased interaction between youth and adults (Coley et al. 1997; Sullivan et al. 2004; Kuo et al. 1998). As with the earlier park movements, these contemporary discussions lack specificity about how parks and other forms of green space promote prosocial behaviour and social cohesion.

We turn, then, to the fields of leisure studies and environmental and social psychology where researchers have focused on the social nature of parks and other natural environments and investigated the implications of these social interactions for different populations across location, age groups, income levels, race/ethnicity, and immigrant status. Rapid urbanization, car ownership, increased employment rates for women, and increased importance of social media and electronic communication has led to the weakening of neighbourhood ties in urban areas (Kazmierczak 2013). Beyond the feelings of security and belonging, neighbourhood social ties may be important to dissemination of information and mutual aid (Kazmierczak 2013; Kuo et al 1998b). Public spaces including neighbourhood parks can facilitate development of meaningful social ties. Kazmierczak (2013) found that even in inner-city neighbourhoods with high levels of deprivation, parks served as sites for initiating and strengthening social ties for those who visit parks regularly. These “everyday places” can contribute to a general sense of well-being by providing a relief from daily routines and stress at home through social interactions that may be as simple as nods and smiles (Cattell et al 2008).

A number of studies have investigated the positive impact of social interactions in parks and other public spaces on facilitating acculturation and adaptation for immigrant groups (Stodolska et al 2016; Peters et al 2010). Main (2013) investigates the meaning of urban parks for immigrants using the concepts of place attachment and place identity, finding that natural and social elements of urban parks can provide important reminders of immigrants’ sending communities. Seeland et al (2009) describe how public urban green spaces can help foster social inclusion as immigrant youths have opportunities to build cross-cultural social capital through sports and other forms of active play.

Several of these studies emphasize the importance of aesthetic qualities and design, arguing that parks need to be attractive and well-maintained, and have adequate seating and shade in order to maximize their positive impacts (Kazmierczak 2013; Francis et al 2012; Peters et al 2010). Preferences regarding park attributes may also differ by gender and ethnicity (Ho et al 2005). Many studies also note that cross-cultural, inter-racial, and inter-ethnic interactions can lead to social tension, particularly in public spaces that may be racially demarcated and where there may be conflict over use of space for activities such as sporting events and vending (Lee and Scott 2013; Main 2013; Peters 2010). Parks must be understood as operating within a historical, socio-ecological, and political-economic context, making them “ideologically charged” and often “ethno-racially inscribed” spaces (Byrne & Wolch 2009) that can be experienced as both barriers (Byrne 2012) or “green walls” (Solecki & Welch 1995), discouraging access, for racial/ethnic minorities, as well as “green magnets” that potentially improve interracial relations (Gobster 1998). In other words, simply facilitating social interactions across groups is not enough to insure positive benefits for immigrants or racial/ethnic minorities.

Allport’s intergroup contact theory (1954) offers a framework for understanding the conditions under which inter-racial and inter-ethnic social interactions, such as those that may occur in urban neighbourhood parks, can have positive impact on people on both sides of the interaction by reducing bias and conflict. These conditions include people across groups experiencing equal group status within the encounter, common goals, an experience of intergroup cooperation, support from authority and “friendship potential” (Brown & Hewstone 2005; Dovidio et al 2003; Pettigrew 1998). Recent research has also considered the role of expectations; when individuals across groups approach intergroup contact with positive expectations, the interactions are more likely to generate positive outgroup attitudes (Deegan et al 2015).

Researchers across disciplines have tested intergroup contact theory in the context of military, worksites, schools, neighbourhoods, housing complexes, and religious congregational settings. Longitudinal studies (Binder et al. 2009, Christ et al. 2010, Eller & Abrams 2004, Levin et al. 2003) and meta-analyses (Pettigrew & Tropp 2008; Hodson & Hewstone 2013) demonstrate consistent and relatively large and positive effects of intergroup contact on prejudice and intergroup conflict across age groups, settings, and countries (Pettigrew 2016). These positive effects of intergroup contact are not limited to the group members who are directly involved in the interaction but extend to the larger group by impacting norms (Pettigrew 2016; Christ et al 2014). Fewer studies have applied intergroup contact theory to urban public places such as neighbourhood parks. One study by Lee and Scott (2013) investigated the experience of Korean American males ages 19–36 playing pickup basketball or soccer. Most participants indicated that interracial contact through recreational sports contributed to harmonious interracial relations and that the optimal conditions of such contact as defined by intergroup theory need not be satisfied for positive contact to occur. They pointed, instead, to skill level and physical attributes of participants, length of contact, and climate within the recreational setting as key factors.

The Lee and Scott (2013) study, like other research in leisure studies focusing on social interactions in public parks, employed a qualitative research methodology (Stodolska et al 2016; Peters 2010; Seeland et al 2009; Cattell et al 2008). While in-depth interviews, ethnography, and focus groups are ideal for understanding the meanings people assign to inter-racial and inter-ethnic interactions, they necessarily employ very small samples that limit their generalizability. Other studies have used surveys to capture information from residents about their inter-racial and inter-ethnic interactions in public parks and the meanings they assign those experiences (Main 2013; Rios et al 2012; Peters et al 2010; Maas et al 2009; Seeland et al 2009; Ho et al 2005). While these studies have larger samples, they rely on self-report about the frequency and conditions of inter-racial and inter-ethnic contact.

### 2.3. Racism and Health Disparities

Within public health literature, concern about the role of urban neighbourhood parks in racial health disparities has focused on lack of physical access to parks, disproportionate exposure to park disamenities, and racial disparities in park use by people of color (Watson et al 2016; Weiss et al 2011; Abercrombie et al 2008). The pathway linking parks and public health has focused on parks as sites for physical activity, not social interaction. Distinct from the extensive literature on parks, public health research has focused on numerous ways in which prejudice and institutional racism negatively impact health, particularly for Blacks/African Americans (Gee et al 2012; Gee 2011; Krieger 1999; Jones 2000). Most research identifies stress, caused by the “accumulated insults arising from every-day and sometimes violent experiences of being treated as a second-class citizen” (Krieger 1999), p 332), as a critical link between racial discrimination and health. Recent research has also documented a connection between discrimination and increased risk-taking behaviours (Jamieson et al 2013). While only one of many aspects of racism, interpersonal conflict and discrimination, or what Krieger (1999) calls “socially inflicted trauma,” contributes to the lived experience—and negative health consequences—of racism. Krieger refers to “embodiment” as the way that discrimination “gets under the skin.” (Krieger 2016; Krieger 1999). Decreasing or eliminating racial prejudice and discrimination could, therefore, have positive health implications for Blacks/African Americans. Numerous studies, most of them published outside of public health, consider parks as sites of racial discrimination (Gobster 2002; Rishbeth 2001; West 1989; Gobster 1989) but they do not link exposure to discrimination to racial health disparities.

### 2.4. Public Health Measures of Park Use and Features

Public health research has focused on the human health implications of parks primarily as sites for promoting physical activity (Lachowycs & Jones 2011; Brownson et al 2009; Kaczynski & Henderson 2008; Cohen et al 2007). While this body of research largely neglects the potential pathway linking parks and improved health through social cohesion and intergroup contact, it does offer important tools for measuring park conditions and activities that can be applied to research focused on these social pathways. Research on parks and physical activity has increasingly employed objective measures of human behaviour, through systematic observation, electronic devices such as accelerometers, heart-rate monitors, and global positioning systems (GPS), and systematic audits of built environment conditions (King et al 2015; McKenzie et al 2006; McKinnon et al 2012). Specifically, studies have employed physical activity logs, GPS, accelerometers, and direct observation of physical activity on the way to (Evenson et al 2013) and within parks (Kaczynski et al 2008; Qigg et al, 2010) to document the public health value of these investments. These are promoted as objective measures of physical activity in response to the documented social desirability and recall bias of survey-based and other self-report measures of physical activity.

In summary, we draw on scholarship from planning and landscape architecture history, leisure studies, psychology and public health to focus attention on the importance of social interactions rather than only on physical activity. In connecting public health research on the negative health impacts of racial discrimination for Blacks/African Americans to Allport’s theory of intergroup contact (1954), we identify a specific possible causal pathway that links positive interracial social interactions in neighbourhood parks to improved health for all groups.

Borrowing from the observation measures used for public health studies on physical activity in parks, we employ a method of systematic observation to identify what combination of people, across age, gender and race, are present in the same section of urban neighbourhood parks across five cities as a first step in understanding the conditions under which inter-racial contact is most likely to occur. By employing quantitative methods to analyse a large sample of observations, we offer a complement to the more nuanced qualitative research in order to identify patterns across multiple cities and parks.

In this study, we address the following research questions: (1) How frequently are people across racial groups present in the same section of parks at the same time? (2) What are the characteristics of park areas and park activities that correlate with the co-presence of park users across racial groups? And (3) What neighbourhood characteristics in which parks are located are correlated with the co-presence of park users across racial groups? We use our results to highlight they ways that urban planners and public health professionals can work deliberately to design and program neighbourhood parks to maximize their public health impact. Our analysis of who is coming into contact in parks has important implications for understanding and, potentially, improving interracial and interethnic relations. This is especially timely in light of the ethnic confrontation that is entangled with contemporary globalization and urbanization around the world, and within the United States, the “Age of Ferguson” and Black Lives Matter movement (Derickson 2016).

## 3. Research Methods

The System for Observing Play and Recreation in the Community (SOPARC) is a validated direct observation tool for assessing use of park and recreation areas, including park users’ physical activity levels, gender, activity types, and estimated age and ethnicity groupings (McKenzie et al. 2006). SOPARC has been used to show variability in physical activity levels across park users by age, gender, race/ethnicity (Besenyi et al. 2013; Reed & Hooker 2012a; Reed et al. 2012; Reed et al. 2011), park areas (Besenyi et al. 2013), parks, cities, and seasons (Cohen et al. 2013; Ward et al. 2014; Chow et al 2016), urban versus rural settings (Shores & West 2010) and neighbourhoods based on walkability, racial composition and income (Dyck et al. 2013; Ward et al 2014; Cohen et al 2013). Previous studies have used SOPARC data on gender, age, and race/ethnicity to document disparities across groups in the use of parks and physical activity levels (Kaczynski et al, 2011; Evenson et al 2016) but not to investigate the combinations of people who are co-present in parks.

Observations were made using SOPARC at 31 neighbourhood parks across five different US cities during the spring, summer and fall between 2008 and 2010. Researchers from Albuquerque, NM, Columbus, OH, Chapel Hill/Durham, NC, and Philadelphia, PA selected six parks each and researchers from Los Angeles CA selected seven neighbourhood parks from areas with different racial/ethnic and income composition. Some but not all parks included a recreation center and full-time staff. Trained staff observed all areas of each park at four randomly-selected 1-hour intervals between 7am and 8pm on two randomly selected weekdays and two randomly-selected weekend days over at least 3 seasons of the year (Cohen et al 2011). The time of day and day of the week were recorded for each observation. Two observers worked together to document the type of activity and each person’s physical activity (sedentary, walking, vigorous), gender, age group (child, adolescent, adult, senior), and race/ethnicity (Latino, African American, White, and other). Reliability checks with a third independent observer were conducted to insure that the procedure had good reproducibility (Ward et al 2014). Prior studies indicate that SOPARC can assess these measures reliably (Cohen et al 2007).

### 3. 1 Characteristics of park target areas

Overall park size was calculated as a continuous variable (in acres). Each park was mapped and divided into discrete target areas to make observations more manageable. The type of facilities present (i.e., playground, baseball field, basketball court, indoor weight room) were documented for each target area. Two staff rotated around the park, systematically observing each target area and identifying whether it was physically accessible (i.e., not locked), empty, organized (scheduled sporting event or exercise class), and supervised by park staff, coach, volunteer, or teacher.

### 3.2 Characteristics of park users

Observations were coded based on the total number of people present, whether at least one male and one female was present, only males were present, or only females were present and whether any children, teens, adults or seniors were present. Based on data collected in the field, all observations were later coded as interracial or not based on whether at least one white and one non-white person were present in the same target area at the same time. Interracial was defined exclusively as the simultaneous presence of someone white and someone non-white in the same target area. We chose to operationalize interracial this way because whites represented the largest (Columbus and Chapel Hill/Durham) or second largest (Albuquerque, Los Angeles, and Philadelphia) demographic group in all of the cities. Also, historically racial/ethnic conflict in the United States has been defined largely in the context of white privilege and white supremacy that categorizes all non-whites as “other” (Mills 1997). Research using SOPARC has consistently shown high levels of inter-rater reliability in regard to the total number of people observed, age, gender, and race/ethnicity (Evenson et al 2016).

### 3.3. Characteristics of activities

Staff identified the number of males and number of females being sedentary or standing without moving (heretofore referred to as sedentary), moderately active (such as walking), or vigorously active. SOPARC has also been shown to have high levels of inter-rater reliability for physical activity levels (Evenson et al 2016). They also identified the primary activity for the females and males inside the target area (i.e., sitting, running, swinging) as well as whether there were any spectators present. All observations were later coded based on whether the primary activity involved a team sport, a playground activity, sedentary activity, such as sitting, standing, picnicking, reading, or lying down, moderate activity such as walking, or whether anyone within the target area was being vigorously active or not.

### 3.4 Characteristics of Park Neighbourhoods

The neighbourhood racial/ethnic and income characteristics of each park were determined using 2000 US Census data for block groups with centroids within half a mile of park boundaries for parks in all cities other than Chapel Hill/Durham where block groups with centroids within 0.8 mile were used because of much lower population densities. The population density and the percent of neighbourhood residents who were white, Black/African American, and Hispanic/Latino and living in poverty were determined for all parks. Neighbourhoods were then identified as having a high interracial mix (no racial/ethnic group made up more than 50% of population) or medium interracial mix (no racial/ethnic group made up more than 70% of the population)., and as having a high poverty (poverty rate greater than 25% or not) or low poverty (poverty rate less than 15% or not). Poverty rate was measured as a continuous variable for the GEE model.

### 3.5 Statistical Analysis

Descriptive statistics were generated to compare the timing of observations, characteristics pf park target areas, and characteristics of park activities across cities and neighbourhoods. Generalized Estimating Equations (GEE) were used to analyse the SOPARC data. GEE models are appropriate given the clustered nature of the sample (i.e., multiple target areas with park) and multiple observations taking place on the same day in the same park in the same city.

This research protocol was approved by the Institutional Review Boards of the University of Pennsylvania, RAND Corporation, The Ohio State University, University of North Carolina, and Behavioral Health Research Center of the Southwest/PIRE.

## 4. Results

Of the 43,706 observations made across the 31 parks, only 7,352 (16.8%) included two or more people present in the same target area at the same time. Less than one-third of these observations (31.6%) included at least one white and one non-white person. The frequently of observations of interracial groups varied by city, with the highest rate (40.5%) in Chapel Hill and the lowest (23.6%) in Philadelphia. There were also significant differences across cities in the age and gender of park participants and the amount of sedentary behavior, walking, vigorous activity, and supervised activity (see Table 1).

Table 2 shows that many of these same variables varied based on the characteristics of neighbourhoods in which the parks were located. The co-presence of park users across racial groups was more likely to occur in neighborhoods with a high inter-racial mix (39.6%) relative to neighbourhoods with a moderate inter-racial mix (28.8%) and in both low poverty (26.2%) and high-poverty (16.3%) areas relative to all areas (31.6%). Differences in who was observed using parks across gender and age-groups was more pronounced based on neighbourhood racial/ethnic and income composition. Female park users were nearly twice as likely to be observed in a target area when no males were present in low-poverty areas (27.3%) than high-poverty areas (13.4%) while male park users were more likely to be observed in a target area with no females present in low-poverty (46.7%) than high-poverty (30.1%) areas. Parks in high-poverty areas were less likely to have any adults (64.3%) or any seniors (4.0%) present than areas overall (80.0% and 11.2%, respectively).

Through the multivariate GEE analysis (Table 3), a number of characteristics of the park users, their activities, and park neighbourhood were significantly associated with the co-presence of park users across racial groups. If children or teens were present or both men and women were present, there was significantly greater likelihood than if only adults, only men, or women were present (log odds ratio = .18, .51, and .62, p=.048, .0001, and .0001, respectively). In terms of what park users were doing, supervised activities were significantly more likely than non-supervised activities (log odds ratio=.79, p=.0001) and vigorous activities (log odds ratio=.42, p=.0001) were significantly more likely than moderate or sedentary activities to involve park users across racial groups. Only 634 of our 7352 observations (8.6%) involved supervised activities, but 358 of these (56.5%) involved the co-presence of people across racial groups and 351 (55.4%) involved at least one person being vigorously active. Gyms, baseball fields, lawns, and tennis courts were most likely to be the sites of supervised activities that included park users across racial groups and vigorous physical activity. Basketball courts were the most likely to be supervised and involve vigorous physical activity, but they were less likely than gyms, baseball fields, and tennis court to have people across racial groups present at the same time.

Neighbourhood characteristics showed some interesting associations, as well. Neighbourhoods with high and medium racial mix were significantly more likely to have park users across racial group co-present (log odds ratio= .72 and .71, p =.009 and .004, respectively) than racially homogenous neighbourhoods in their community parks. Poverty level of a neighbourhood had a significant and complex relation, through the interaction with the percentage of white population in the neighbourhood. In a white-majority neighbourhood (e.g., %white = 50%), poverty level was not significantly associated with inter-racial grouping. In a neighbourhood with a relatively low percent of white residents (e.g., %white=10%), poverty level had a significant and negative association (log odds ratio = −.05, p=.01). On the other hand, the percentage of white population always had a significant and positive association regardless of the local poverty level. For example, in a relatively high-income neighbourhood with 10% households in poverty, every percentage point of white population had an estimated log odds ratio of .02 (p=.0004) for inter-racial grouping. In a relatively low-income neighbourhood with 30% households in poverty, the log odds ratio for inter-racial grouping is .04 (p=.0001). Differences among cities were not significant in the multivariate model when controlling for the percent poverty and racial composition of the area around the park, suggesting the lack of unobserved confounders for the outcome of interest besides poverty, racial/ethnicity structure, and their interaction.

## 5. Discussion

Extensive observation across five cities, three seasons, and 31 neighborhoods reveal that only a fraction of target areas in neighbourhood parks are populated by two or more people at any given time, and in less than one-third of the populated areas those park users represented different racial groups, defined as at least one white and one non-white person. Still, we identified 2123 instances where people of different racial groups were co-present, suggesting that neighbourhood parks can potentially serve as places that promote intergroup contact. While our results speak only to co-presence, and not necessarily “contact” as described by Allport, they provide some quantitative evidence that applying interracial contact theory to understanding psychosocial pathways between park use and human health is worthwhile. Furthermore, our research shows that certain parks, park users, and neighbourhood characteristics make the co-presence of park users across racial groups—and potentially interracial contact—more or less likely. Some of these, like neighbourhood racial/ethnic and income composition, cannot be changed easily, while others, such as whether males and females and children are present at the same time or activities are supervised, are modifiable. To understand the impact of these empirical results on intergroup contact, we turn first to the modifiable factors where there are the greatest opportunities for intervention.

The factor that can potentially be modified most easily is the supervision of specific activities in parks. Supervision might take the form of a coach, referee, park staff person, or an adult who represents some level of authority and provides a certain amount of oversight. While having full-time staff at neighbourhood parks may be financially unrealistic in all communities, volunteers including summer high school and college interns, graduate students and faculty (Han et al 2015), City Year and VISTA (Volunteers in Service to America) volunteers, or retirees may present low- to no-cost strategies for organizing and supervising activities in neighbourhood parks. This could be modelled after supervised recess at school through programs such as Playworks (Beyler et al 2014)

Unlike the supervision of park activities, neighbourhood racial/ethnic and income composition—which also holds a considerable influence on whether people across racial groups are co-present—are not easily modifiable. The neighbourhood parks with the most observations including people across racial groups were located in areas of relatively low poverty and majority but not exclusively white populations. The one exception was a park in Los Angeles that had a moderate poverty rate (18.3%) and no majority racial/ethnic population but significant white and Latino populations. The neighbourhood parks with the highest poverty rates and largest Black/African American populations were least likely to have people across racial groups co-present. This does not preclude interventions focused on increasing the amount of supervised activities in neighbourhood parks; having supervised activities makes vigorous physical activity more likely even when interracial contact is unlikely. But deliberate efforts to promote interracial contact are most likely to be successful in areas of low and moderate poverty and with at least some racial/ethnic mix. That parks in areas with even non-majority Black/African-American populations are unlikely to have much interracial contact demonstrates the high levels of white prejudice that need to be reversed. These results demonstrate yet another way that the persistence and co-occurrence of racial/ethnic and income segregation at the neighbourhood level can reinforce health disparities by making intergroup contact in parks unlikely (Krieger et al 2016).

The strengths of this research include the large sample of observations from neighbourhood parks across five different cities and different racial/ethnic and income composition. No previous published study has analysed such a large number of observations as an objective measure of the co-presence of racial groups. While the parks were not selected at random, the days of the weeks and times of the day when observations were conducted were selected randomly, and observations were conducted over three seasons, depending upon the city, allowing for some generalizability of findings across US cities.

The limitations of this analysis are important to acknowledge. Operationally defining interracial as involving white and non-white park users likely underestimates the true amount of interracial activity, which could include co-presence in park areas among non-white groups such as Asians, Blacks/African Americans, and Latinos that could also have important health implications. This binary approach to defining interracial also masks important historical differences in how Hispanic/Latinos and Asians are perceived and treated by whites as forms of discrimination between minority groups (Fernandez & Witt 2013; Sharaievska et al, 2010). Using SOPARC, we are able to identify areas where people across racial groups are co-present, but we cannot assume this involved contact, as described by Allport. In reality, people occupying the same general area within a park could be participating in separate activities that involve no interaction. Also, our analysis treats individual park target areas as the unit of analysis without accounting for their size.

## 6. Implications for Future Research

Research on intergroup contact frequently emphasizes the conditions that facilitate positive effects, such as the presence of authority to support both groups. We may be able to infer that supervision of park activities constitutes this authority, but we know nothing about the nature of those interactions—including the amount of civility, engagement, friendship, or conflict—through observations using the conventional SOPARC measure. Further research is needed to investigate the nature of the interactions among people across racial groups and their impact on individual attitudes and behaviours. Adaptations to SOPARC might include new considerations of the verbal language, body language, tone of voice, eye contact, physical contact, and other characteristics of the interaction among park users. Or, on the model of Cohen et al, 2016, separate measures of intoxication, smoking, fighting, or groups of people who were intimidating others within parks might be used in conjunction with SOPARC observations to measure conflict and potentially negative interactions. Existing measures of segregation might also be applied to the spatial configuration of park users across race/ethnicity, on the model of Echols et al (2014) study of seating patterns in a school cafeteria using measures of exposure (potential for interaction among people across groups) and entropy (how evenly people across group are spread out over a space).

This study calls on researchers across disciplines to consider more broadly the contributions of parks to public health beyond physical activity and the psychosocial benefits of exposure to nature. Urban parks were purposefully designed in the 19^th^ Century with high expectations and democratic ideals, even if they may not have been intended to challenge white prejudice and institutional racism. The potential for neighbourhood parks and other outdoor, green public spaces to promote interracial contact represents an important and underappreciated pathway linking urban design and human health. Urban planning must meet this mandate for realizing the promise of neighbourhood parks by more carefully theorizing, designing, maintaining and then activating these public spaces to achieve equity and health.

## Figures and Tables

**Table 1:** Descriptive Statistics by City for Sample of Park Observations (N=7352*)

	All cities N=7352	Albuquerque N=1141	Chapel Hill/ Durham NC N=1664	Columbus OH N=826	Los Angeles N=2193	Philadelphia N=1528
***Timing of Observations***						

weekend	3509 (47.7%)	628 (55.0%)	1042 (62.6%)	403 (48.8%)	733 (33.4%)	703 (46.0%)
Spring	1777 (24.2%)	458 (40.1%)	583 (35.0%)	233 (28.2%)	0**	503 (32.9%)
Summer	3085 (42.0%)	353 (30.9%)	459 (27.6%)	330 (40.0%)	1339 (61.1%)	604 (39.5%)
Fall	2248 (30.6%)	330 (28.9%)	622 (37.4%)	263 (31.8%)	612 (27.9%)	421 (27.6%)

***Characteristics of Target Areas***						

playground	897 (12.2%)	145 (12.7%)	192 (11.5%)	129 (15.6%)	209 (9.5%)	222 (14.5%)
supervised	634 (8.6%)	30 (2.6%)	193 (11.6%)	113 (13.7%)	229 (10.4%)	69 (4.5%)
team sport	885 (12%)	108 (9.5%)	157 (9.4%)	83 (10.0%)	306 (14.0%)	231 (15.1%)

***Characteristics of Park Activities and People in Target Areas***						

interracial	2321 (31.6%)	387 (33.9%)	674 (40.5%)	245 (29.7%)	655 (29.9%)	360 (23.6%)
Physical Activity:						
sedentary	2353 (32.0%)	367 (32.2%)	313 (18.8%)	172 (20.8%)	946 (43.1%)	555 (36.3%)
walking	998 (13.6%)	223 (19.5%)	294 (17.7%)	41 (5.0%)	288 (13.1%)	152 (9.9%)
vigorous	2654 (36.1%)	285 (25.0%)	761 (45.7%)	323 (39.1%)	767 (35.0%)	518 (33.9%)
Gender***:						
male and female	3549 (48.3%)	285 (25.0%)	761 (45.7%)	323 (39.1%)	767 (35.0%)	518 (33.9%)
female only	890 (12.1%)	151 (13.2%)	143 (8.6%)	68 (8.2%)	261 (11.9%)	267 (17.5%)
male only	1526 (20.8%)	211 (18.5%)	254 (15.3%)	131 (15.9%)	586 (26.7%)	344 (22.5%)
Age Group:						
any children	4060 (55.2%)	609 (53.4%)	947 (56.9%)	590 (71.4%)	1115 (50.8%)	799 (52.3%)
any teens	1791 (24.4%)	261 (22.9%)	284 (17.1%)	271 (32.8%)	555 (25.3%)	420 (27.5%)
any adults	5885 (80.0%)	922 (80.8%)	1491 (89.6%)	559 (67.7%)	1924 (87.7%)	989 (64.7%)
any seniors	820 (11.2%)	163 (14.3%)	240 (14.4%)	36 (4.4%)	333 (15.2%)	48 (3.1%)

* This represents the subset of all observations where two or more people were present in the same park target area at the same time.

** No observations were conducted during the spring in Los Angeles.

*** These values do not add up to 100% because of missing data on gender.

**Table 2:** Descriptive Statistics by Area Racial/Ethnic and Income Composition for Sample of Park Observations

	Interacial mix			Poverty
	Moderate interracial N=2,420	High interracial N=1,183	Low poverty N=3266	High poverty N=1269
weekend	1242 (51.3%)	399 (33.7%)	1610 (49.3%)	515 (40.6%)
Spring	743(30.7%)	175 (14.8%)	573 (17.5%)	288 (22.7%)
Summer	1110 (45.9%)	840 (71.0%)	947 (29.0%)	646 (50.9%)
Fall	567 (23.4%)	97 (8.2%)	1643 (50.3%)	335 (26.4%)

playground	335 (13.8%)	169 (14.3%)	315 (9.6%)	160 (12.6%)
supervised	98 (4.0%)	85 (7.2%)	254 (7.8%)	101 (8.0%)
team sport	235 (9.7%)	187 (15.8%)	628 (19.2%)	238 (18.8%)

interracial	698 (28.8%)	469 (39.6%)	856 (26.2%)	207 (16.3%)
Physical Activity:				
sedentary	891 (36.8%)	524 (44.3%)	921 (28.2%)	458 (36.1%)
walking	296 (12.2%)	149 (12.6%)	484 (14.8%)	108 (8.5%)
vigorous	706 (29.2%)	396 (33.5%)	1207 (37.0%)	427 (33.6%)
Gender:				
male and female	1239 (51.2%)	659 (55.7%)	452 (35.6%)	452 (35.6%)
female only	320 (13.2%)	153 (12.9%)	890 (27.3%)	170 (13.4%)
male only	439(18.1%)	303 (25.6%)	1526 (46.7%)	382 (30.1%)
Age Group:				
any children	1325 (54.8%)	649 (54.9%)	1428 (43.7%)	665 (52.4%)
any teens	570 (23.6%)	349 (29.5%)	694 (21.2%)	501 (39.5%)
any adults	1875 (77.5%)	997 (84.3%)	2551 (78.1%)	816 (64.3%)
any seniors	231 (9.5%)	123 (10.4%)	369 (11.3%)	510 (4.0%)

**Table 3:** Analysis Of GEE Parameter Estimates for Interracial Contact*

	Estimate	SE	95% Confidence Interval		Z-value	p-value
***State***						

CA	0.198	0.3135	−0.4165	0.8126	0.63	0.5277
NC	0.3877	0.2934	−0.1873	0.9626	1.32	0.1864
NM	0.3121	0.3101	−0.2957	0.9198	1.01	0.3143
OH	0.3452	0.3265	−0.2947	0.9851	1.06	0.2903
PA	referent	referent	referent	referent	referent	referent

***Timing of observation***						

Weekend	−0.1982	0.0996	−0.3935	−0.0029	−1.99	0.0466
Spring	−0.0515	0.0875	−0.2229	0.1199	−0.59	0.5559
Summer	−0.0835	0.0641	−0.2091	0.0421	−1.3	0.1928
Fall	referent	referent	referent	referent	referent	referent

***Characteristics of Park and Target Areas***						

park size (acres)	0.0015	0.0079	−0.014	0.0171	0.19	0.8464
playground	−0.1649	0.1349	−0.4293	0.0995	−1.22	0.2216
team sport	0.1228	0.0983	−0.0698	0.3154	1.25	0.2115
supervised	0.7927	0.1157	0.5659	1.0196	6.85	<.0001

***Characteristics of Park Activities and People in Target Areas***						

Physical Activity:						
sedentary	−0.0175	0.0647	−0.1443	0.1093	−0.27	0.787
walking	−0.1522	0.1189	−0.3851	0.0808	−1.28	0.2005
vigorous	0.4166	0.0641	0.2909	0.5424	6.5	<.0001
Gender:						
male and female	0.6184	0.0749	0.4715	0.7652	8.25	<.0001
female only	−0.1128	0.1348	−0.377	0.1514	−0.84	0.4027
Male only	Referent	Referent	Referent	Referent	Referent	referent
Age Group:						
any children	0.1836	0.0928	0.0017	0.3655	1.98	0.0479
any teens	0.514	0.0777	0.3617	0.6663	6.61	<.0001

***Characteristics of Park Neighborhood***						

percent poverty	−0.0573	0.0224	−0.1011	−0.0134	−2.56	0.0104
percent white	0.0135	0.0094	−0.005	0.032	1.44	0.1513
%pov * %white	0.0009	0.0004	0.0001	0.0016	2.33	0.02
high racial mix	0.7183	0.2751	0.1792	1.2575	2.61	0.009
mod racial mix	0.7129	0.248	0.2268	1.199	2.87	0.004

* Statistical model adjusts for everything listed in the table in addition to accounting for the correlation of multiple target areas within parks.
